# EXPLORING EXPERIENCES OF PAIN MANAGEMENT AMONG FAMILY CAREGIVERS OF COMMUNITY-DWELLING OLDER ADULTS WITH DEMENTIA

**DOI:** 10.1093/geroni/igac059.016

**Published:** 2022-12-20

**Authors:** Hui Zhao, Pamela Kulbok, Ishan Williams, Carol Manning, Rafael Romo

**Affiliations:** James Madison University, Harrisonburg, Virginia, United States; UVA, Charlottesville, Virginia, United States; University of Virginia, School of Nursing, Charlottesville, Virginia, United States; UVA, Charlottesville, Virginia, United States; Dominican University of California, San Rafael, California, United States

## Abstract

Pain is often under reported and under-treated in older adults with dementia. Formal caregivers receive training and resources to develop their pain management skills; yet family caregivers (FCGs), who bear the brunt of responsibility for pain management among community-dwelling older adults with dementia have largely been omitted from research. We conducted a qualitative descriptive study to gain a deep understanding of FCGs’ experience in pain management. 25 adult FCGs of community-based older adults with dementia were living in central Virginia were interviewed. Participants were 29 to 95 years old, predominantly white, married, female, and high school graduates. Four themes emerged around exploring FCGs’ pain management experience and each theme included sub themes: 1) Values: family caregivers make values-based decisions that rely on a diverse range of beliefs towards opioids and non-pharmacological approaches. 2) Barriers: pain management was hampered by patient-related factors (comorbidity, complexity of care) and FCG-related factors (lack of training and resources). 3) Support: FCGs perceived greater competence when well supported by professional caregivers (doctors, social workers) and family members. 4) Adaptation: FCGs employed many strategies to support themselves and build a sense of self-efficacy that can either inhibit or facilitate effective pain management for their loved-ones. Adaptation and support from professional or formal caregivers greatly improved FCGs’ perception of competence in pain management, suggesting research and development of interventions targeting FCGs is warranted.

